# The Feasibility and Effectiveness of Web-Based Advance Care Planning Programs: Scoping Review

**DOI:** 10.2196/15578

**Published:** 2020-03-17

**Authors:** Doris van der Smissen, Anouk Overbeek, Sandra van Dulmen, Lisette van Gemert-Pijnen, Agnes van der Heide, Judith AC Rietjens, Ida J Korfage

**Affiliations:** 1 Department of Public Health Erasmus MC, University Medical Center Rotterdam Rotterdam Netherlands; 2 Department of Criminal Law Erasmus University Rotterdam Rotterdam Netherlands; 3 Nivel (Netherlands institute for health services research) Utrecht Netherlands; 4 Department of Primary and Community Care Radboud Institute for Health Sciences Radboud university medical center Nijmegen Netherlands; 5 Faculty of Health and Social Sciences University of South-Eastern Norway Drammen Norway; 6 Centre for eHealth and Wellbeing Research Department of Psychology, Health and Technology University of Twente Enschede Netherlands

**Keywords:** advance care planning, Web-based intervention, decision aids, patient education, eHealth, health communication, patient-centered care, chronic disease

## Abstract

**Background:**

Advance care planning (ACP) is a process with the overall aim to enhance care in concordance with patients’ preferences. Key elements of ACP are to enable persons to define goals and preferences for future medical treatment and care, to discuss these with family and health care professionals, and to document and review these if appropriate. ACP is usually conducted through personal conversations between a health care professional, a patient, and—if appropriate—family members. Although Web-based ACP programs have the potential to support patients in ACP, their effectiveness is unknown.

**Objective:**

This study aimed to assess the feasibility and effectiveness of Web-based, interactive, and person-centered ACP programs.

**Methods:**

We systematically searched for quantitative and qualitative studies evaluating Web-based, interactive, and person-centered ACP programs in seven databases including EMBASE, Web of Science, Cochrane Central and Google Scholar. Data on the characteristics of the ACP programs’ content (using a predefined list of 10 key elements of ACP), feasibility, and effectiveness were extracted using a predesigned form.

**Results:**

Of 3434 titles and abstracts, 27 studies met the inclusion criteria, evaluating 11 Web-based ACP programs—10 were developed in the United States and one in Ireland. Studied populations ranged from healthy adults to patients with serious conditions. Programs typically contained the exploration of goals and values (8 programs), exploration of preferences for treatment and care (11 programs), guidance for communication about these preferences with health care professionals or relatives (10 programs), and the possibility to generate a document in which preferences can be recorded (8 programs). Reportedly, participants were satisfied with the ACP programs (11/11 studies), considering them as easy to use (8/8 studies) and not burdensome (7/8 studies). Designs of 13 studies allowed evaluating the effectiveness of five programs. They showed that ACP programs significantly increased ACP knowledge (8/8 studies), improved communication between patients and their relatives or health care professionals (6/6 studies), increased ACP documentation (6/6 studies), and improved concordance between care as preferred by the patients and the decisions of clinicians and health care representatives (2/3 studies).

**Conclusions:**

Web-based, interactive, and person-centered ACP programs were mainly developed and evaluated in the United States. They contained the key elements of ACP, such as discussing and documenting goals and preferences for future care. As participants considered programs as easy to use and not burdensome, they appeared to be feasible. Among the 13 studies that measured the effectiveness of programs, improvement in ACP knowledge, communication, and documentation was reported. The concordance between preferred and received care is yet understudied. Studies with high-quality study designs in different health care settings are warranted to further establish the feasibility and effectiveness of Web-based ACP programs.

## Introduction

### Background

Contemporary conceptualization of advance care planning (ACP) defines ACP as a process that enables persons to define goals and preferences for future medical treatment and care and to discuss these with family and health care professionals [1]. Furthermore, persons may record and review these preferences if appropriate [1]; therefore, preferences can be accessed when these persons are not able to speak for themselves. The overall aim of ACP is to improve concordance between preferred and received care. ACP may be useful in any stage of life but becomes more targeted when a person’s health condition worsens or when age increases [1]. ACP is usually conducted through a structured, personal conversation between a health care professional, a patient, and—if appropriate—family members. Since the 1990s, evidence has amassed, showing that ACP interventions have potentially beneficial outcomes for patients and health care systems. These include increased completion of advance directives (ADs): documents in which preferences for future medical treatment and care can be recorded [2,3]. Furthermore, these beneficial outcomes include better alignment of care to expressed preferences, better quality of communication in clinical consultations, improved quality of life, reduction of unwanted hospital admissions, and increased use of palliative care [2,3].

Health care professionals and patients generally underline the importance of ACP [4]. Given that the number of people with chronic conditions is increasing [5] and that ACP can be relevant in the early stages of disease, ACP will become relevant for a growing number of people. Nevertheless, the implementation of ACP in practice faces several challenges [4,6,7]. The (facilitated) ACP process takes time, and supporting patients in this process is, therefore, costly [4,6]. This limits the upscaling of and accessibility to the ACP process. Engagement in ACP is further limited by the delay in its initiation because of the barriers experienced by health care professionals and patients: health care professionals report concerns about taking away patients’ hope and uncertainty about timing of ACP, whereas patients expect health care professionals to initiate ACP [4,6]. Further barriers to engagement in ACP are physicians’ lack of training in having ACP conversations and lack of continuity of care [4,6]. Furthermore, people with chronic diseases may not have the time and energy for face-to-face conversations, for example, because of treatment burden, even if these conversations would help them [8-10]. Still, there are patients and healthy individuals who experience a clear need to engage in ACP. For instance, 398 of 502 (79.3%) Belgian citizens aged 64 years and older indicated to be willing to take the initiative to start the ACP process, for example, by completion of an AD [11].

One way of overcoming the barriers to wider implementation of ACP may be Web-based ACP programs [12-14]. They can be accessed on the Web at any preferred time, have the potential to reach a larger audience, are relatively easy to implement, and are scalable. Moreover, a Web-based format of ACP may be an addition to the ACP process as facilitated by professionals, as it can be delivered stepwise and tailored and can include interactive elements and videos. Web-based ACP programs should not replace discussions with health care professionals or with ACP facilitators, but they may support patients to prepare these discussions and to consider their values, beliefs, and care preferences in their own time and environment. Ample research in other domains has shown that Web-based health programs can be effective in improving health outcomes such as physical activity [15], patient empowerment [15,16], and depression [17]. They have the potential to be cost-effective [18]. Patients perceive Web-based health programs usually to be useful and helpful [19]. Therefore, Web-based ACP programs may have the potential to support patients in ACP.

Several reviews described person-centered tools, including decision aids targeted at adult patients and their relatives as well as healthy individuals. These tools are related to ACP, shared decision making, and end-of-life care. Some studies in these reviews also included Web-based or computerized programs [14,20-24]. None of these reviews focused specifically on empirically evaluated, Web-based, and available programs for ACP and their feasibility and effectiveness. In addition, none of the reviews focused on interactive programs, which guide users through the information and in which users are enabled to interact with the information. Only Butler et al [14] focused specifically on ACP decision aids. Most of the reviews (except for the study by Butler et al [14]) focused on specific populations instead of providing an overview of available ACP programs for the general population as well as for patients.

### Scope of This Review

The overall aim of this scoping review was to assess the feasibility and effectiveness of Web-based, interactive, and person-centered ACP programs. This review focuses on the following research questions: (1) What are the functionalities of Web-based ACP programs?, (2) What is the content of Web-based ACP programs?, (3) How feasible are Web-based ACP programs?, and (4) How effective are Web-based ACP programs?

## Methods

### Methodological Framework

Scoping studies “aim to map rapidly the key concepts underpinning a research area and the main sources and types of evidence available and can be undertaken as stand-alone projects in their own right, especially where an area is complex or has not been reviewed comprehensively before” [25]. Scoping reviews can be used to explore the literature within a research area of interest by addressing broad research questions. This exploration can be done regardless of the methodological quality of the studies or risk of bias [26,27]. We used a systematic approach, namely, the methodological framework for scoping reviews by Arksey and O’Malley [26]. The five stages of the framework for scoping reviews are (1) identifying the research question; (2) identifying relevant studies; (3) study selection; (4) charting the data; and (5) collating, summarizing, and reporting the results [26].

### Search Strategy

The search strategy was developed in collaboration with a medical librarian. We systematically searched for empirical studies written in the English language that evaluated Web-based, interactive, and person-centered ACP programs. We searched in EMBASE on July 24, 2017, and in Medical Literature Analysis and Retrieval System Online Epub (MEDLINE Epub [Ovid]), Web of Science, Cochrane Central, PsycINFO (Ovid), Cumulative Index to Nursing and Allied Health Literature (CINAHL [EBSCO]), and Google Scholar on July 28, 2017, and updated this search on April 16, 2019. Multimedia Appendix 1 presents the search strategy.

### Study Selection

Duplicates were removed. Two reviewers (DS and AO) independently screened titles, abstracts, and full text of articles to identify relevant studies, assisted by the program Covidence (operated by Veritas Health Innovation Ltd) [28]. Articles were included when they fulfilled the inclusion criteria, as presented in Textbox 1. In addition, we handsearched the references of included articles and other possibly relevant articles. When DS and AO could not reach consensus about inclusion or exclusion, other authors were consulted (IK and JR). Disagreements were readily resolved.

**Inclusion criteria for the full-text papers.**

1. The study has an original empirical quantitative or qualitative research design. Reviews and conference abstracts were excluded.

2. The study evaluates a program that:

- supports the completion of one or more elements of advance care planning (ACP), defined as enabling persons to define, discuss, record, and review goals and preferences for future medical treatment and care [1];
- is accessible and available on the internet;
- is interactive, defined as guiding users through the ACP process in which they are enabled to interact with information instead of only reading text; and
- is person centered, defined as being targeted at adult patients, relatives, and/or healthy individuals in general rather than solely at clinicians or medical students.

3. Language of the publication should be English.

### Data Extraction and Outcomes of Interest

Data extraction was performed by DS and AO using a predesigned form. Data were extracted from the ACP programs and from the studies evaluating the ACP programs.

#### Advance Care Planning Programs

Functionalities of the ACP programs were extracted based on the Consolidated Standards of Reporting Trials of Electronic and Mobile HEalth Applications and onLine TeleHealth (CONSORT-EHEALTH) checklist [29], which is developed to ensure that electronic health (eHealth) interventions in randomized controlled trials (RCTs) are reported in sufficient detail for replication. We extracted the programs’ target group and accessibility, for example, whether it was possible to access the program without registration. Furthermore, we extracted whether the programs were free of charge; were tailored to the users’ information needs; provided feedback on responses; showed progress information; had the possibility of giving input, for example, to answer questions; contained hyperlinks to (external) Web pages; contained a text-to-speech option; contained videos; could be used without assistance; addressed the privacy policy; and addressed log data analysis (tracking behavior of users in a Web-based program).

The content of the ACP programs was extracted based on the European Association for Palliative Care (EAPC) consensus definition of ACP [1]. In this review, the EAPC ACP task force defined 12 key elements of ACP, which we summarized into 10 elements, such as providing information about ACP, addressing the readiness/timing for ACP, addressing exploration of values and goals, and addressing recording of ACP and ACP communication [1].

#### Advance Care Planning Studies

The following study characteristics were extracted: first author and year, country, participants and setting, study design, intervention and outcome measures, and results of the studies on feasibility and effectiveness of ACP programs.

The feasibility of the ACP programs was extracted based on the framework of Bowen et al [30]. We extracted the acceptability of the burden of the program, ease of use, understandability of the text in the program, and the acceptability of the program. To briefly address the implementation of the programs, we extracted whether further developments or research of the programs were described. Furthermore, we extracted outcomes as recommended by the CONSORT-EHEALTH checklist [29], namely, participation rates among the contacted participants, completion rates of those who provided consent completing the entire program, whether the use of log data of users was described, and whether user feedback was obtained.

To report on the effectiveness of ACP programs, we used the outcome measures of ACP that were recommended by the EAPC ACP task force, such as ACP knowledge; self-efficacy; identification of goals, values, and preferences; helpfulness in ACP (for making decisions); health care use; and decision concordance between the patients’ preferences and health care professionals’ decisions [1].

## Results

### Inclusion of Papers

**Figure 1:**
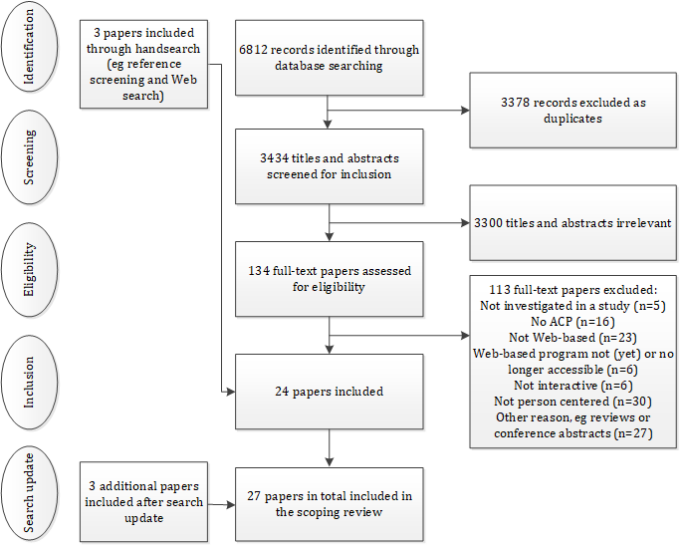
Flowchart of the inclusion of papers.

The search resulted in 6812 records (see Figure 1). After removing duplicates, 3434 titles and abstracts remained. On the basis of the inclusion criteria, 3300 titles and abstracts were found to be irrelevant and were excluded. Next, 134 studies were screened full text, of which 113 studies were excluded (see Figure 1 for details on exclusion). Twenty-one studies were identified as relevant. Handsearch of systematic reviews and potentially relevant other references resulted in the inclusion of three further studies. Overall, in 2017, 24 studies were included for data extraction. On the basis of their initial independent scoring, DS and AO had an agreement for 110 of the 134 full texts (82.1%) about inclusion or exclusion. The interrater reliability is considered moderate (kappa=0.52). Disagreements about inclusion or exclusion were readily resolved, and it was seldom necessary to consult other authors.

In 2019, 983 new references were identified, of which 36 were screened for full-text review. Three articles were included, which analyzed two programs that were already described in this review. This resulted in a total number of 27 included articles.

### Functionalities of the Web-Based Advance Care Planning Programs

Table 1 presents an overview of the functionalities of the Web-based ACP programs, and Multimedia Appendix 2 presents the links to the Web-based programs. The 27 included studies evaluated 11 Web-based programs—8 programs were targeted at patients or healthy individuals, two programs were targeted at patients and relatives, and one program was targeted at patients and health care professionals. Related to the accessibility of the Web-based programs, we found that six programs were accessible without registration, and 10 programs were free of charge. Ten programs could be used without assistance of a health care professional. Related to the interactivity, we found that all programs provided the possibility for the users to give input, for example, by answering questions. Eight programs included an indicator of the progress of users in completing the program. Seven programs could tailor to users’ information needs by providing additional information if preferred, and six programs contained hyperlinks to (external) Web pages. Three of the programs provided (specific) feedback on responses of users, for example, by giving a personalized response to answered questions in the program. Related to the layout, eight programs contained videos, and two had a text-to-speech option to play text in audio. Finally, eight programs described their privacy policy in the program and reported that they analyzed log data of users. The program PREPARE For Your Care (PREPARE) addresses 11 of 12 functionalities, and the program Making Your Wishes Known addresses 10 functionalities. The programs Death over Dinner, MyDirectives, and Think Ahead address nine functionalities each, and all other programs address five to eight functionalities.

**Table 1:** Functionalities of the Web-based advance care planning programs.

Programs	Target group	Accessible (without registration)	Free of charge	Can be used without assistance	Possibility to give input (eg, answer questions)	Shows progress information	Tailored to users’ information needs	Contains hyperlinks to (external) Web pages	Provides feedback on responses	Contains videos	Text-to-speech option	Privacy policy addressed	Log data analysis addressed
ACP Decisions [31]	P^a^ and H^b^	x^c^	✓^d^	x	✓	x	x	x	x	✓	x	✓	✓
Death over Dinner [32]	P and R^e^	✓	✓	✓	✓	✓	✓	✓	✓	✓	x	x	x
Five Wishes [33]	P	x	x	✓	✓	✓	x	✓	x	✓	x	✓	✓
Making Your Wishes Known [34-45]	P	x	✓	✓	✓	✓	✓	x	✓	✓	✓	✓	✓
MyDirectives [38,46]	P	x	✓	✓	✓	✓	✓	✓	x	✓	x	✓	✓
MyICUGuide [47]	P and R	✓	✓	✓	✓	✓	x	x	x	x	x	✓	✓
NVLivingWill [48]	P	x	✓	✓	✓	✓	✓	✓	x	x	x	x	x
Plan Your Lifespan [49]	P	✓	✓	✓	✓	✓	✓	✓	x	✓	x	x	x
PREPARE For Your Care [38,50-55]	P	✓	✓	✓	✓	✓	✓	x	✓	✓	✓	✓	✓
The Letter Project Advance Directive [56]	P	✓	✓	✓	✓	x	x	x	x	x	x	✓	✓
Think Ahead [57]	P	✓	✓	✓	✓	x	✓	✓	x	✓	x	✓	✓
Total^f^	-	6	10	10	11	8	7	6	3	8	2	8	8

^a^P: patients.

^b^H: health care professionals.

^c^x: not addressed in the program.

^d^✓: addressed in the program.

^e^R: relatives.

^f^Total number of programs that addressed the functionalities.

### Content of the Web-Based Advance Care Planning Programs

Table 2 presents an overview of the content of the Web-based ACP programs, and Multimedia Appendix 2 presents the links to the Web-based programs. Target groups, for example, patients, were involved in the development of seven programs [31,34-45,47,49-57]. Four programs had a theory base [34-45,47,50,57], for example, Making Your Wishes Known was based on the Multi-Attribute Utility Theory, and PREPARE was based on behavior change theories. Related to the key elements for ACP, we found that almost all programs provided information about ACP (10 programs) and included attention for readiness for ACP or for adequate timing of ACP (10 programs). Furthermore, the exploration of goals and values for future treatment and care was addressed by eight programs. In all programs, attention was paid to treatment and care options and treatment and care preferences. Furthermore, all programs addressed the potential appointment of a health care representative (ie, someone who can make decisions on behalf of the patient when he or she is unable to do so) and paid attention to the recording of ACP: eight programs included the possibility to generate a document in which patients can record their goals, values, and preferences. In nine programs, users were encouraged to share this document with their relatives or health care professionals. Ten programs addressed how to communicate preferences with health care professionals or with relatives.

**Table 2:** Inclusion of the recommended key elements for advance care planning in the Web-based advance care planning programs.

Programs	Provides information about ACP^a^	Addresses readiness/timing for ACP	Addresses exploration of values/goals	Addresses treatment and care options	Addresses treatment and care preferences	Addresses appointment of a health care representative	Addresses recording of ACP	Generates document	Encourages to share the document	Addresses ACP communication
ACP Decisions [31]	✓^b^	✓	✓	✓	✓	✓	✓	x^c^	x	✓
Death over Dinner [32]	✓	x	x	✓	✓	✓	✓	x	✓	✓
Five Wishes [33]	✓	✓	✓	✓	✓	✓	✓	✓	✓	✓
Making Your Wishes Known [34-45]	✓	✓	✓	✓	✓	✓	✓	✓	✓	✓
MyDirectives [38,46]	✓	✓	✓	✓	✓	✓	✓	✓	✓	✓
MyICUGuide [47]	✓	✓	✓	✓	✓	✓	✓	✓	✓	✓
NVLivingWill [48]	✓	✓	x	✓	✓	✓	✓	✓	✓	✓
Plan Your Lifespan [49]	✓	✓	x	✓	✓	✓	✓	✓	x	✓
PREPARE For Your Care [38,50-55]	✓	✓	✓	✓	✓	✓	✓	✓	✓	✓
The Letter Project Advance Directive [56]	x	✓	✓	✓	✓	✓	✓	✓	✓	x
Think Ahead [57]	✓	✓	✓	✓	✓	✓	✓	x	✓	✓
Total^d^	10	10	8	11	11	11	11	8	9	10

^a^ACP: advance care planning.

^b^✓: addressed in the program.

^c^x: not addressed in the program.

^d^Total number of programs that addressed the elements.

### Evaluation of the Web-Based Advance Care Planning Programs

Most programs were evaluated in one study [31-33,47-49,56,57], two studies evaluated MyDirectives [38,46], seven studies evaluated PREPARE [38,50-55], and 12 studies evaluated Making Your Wishes Known in [34-45]. Multimedia Appendix 3 presents an overview of the characteristics of the studies. All programs were developed in the United States, except for Think Ahead, which was developed in Ireland [57]. All studies were published in the period from 2007 to 2018, of which nine studies were published in 2017. In total, 25 of the studies have a quantitative design [31,33-49,51-57], and two study designs are qualitative [32,50]. Nine studies allowed comparison of outcomes before/after an intervention [31,35,40,42,43,45,53-55], and eight studies allowed comparison between intervention and control groups [31,39,45,49,52,54-56]. Studied populations ranged from healthy adults to patients with serious conditions. The sample sizes of the quantitative studies ranged from 17 to 3119, and participation rates ranged from 14% to 100%. The use of validated measures, if applicable, is indicated in Multimedia Appendix 3.

### Feasibility of the Web-Based Advance Care Planning Programs

Multimedia Appendix 4 presents an overview of the feasibility of the Web-based ACP programs of the 25 quantitative studies. The participation rate among contacted participants was over 60% in six studies and ranged from 14% to 58% in 12 studies. Seven studies did not report on participation rates. The completion rate considering the entire program ranged from 31% to 72% in five studies and ranged from 83% to 100% in the other 17 studies. Three studies did not report on completion rates. One paper used log data analysis [58] to assess feasibility, seven studies obtained (qualitative) user feedback, and 12 studies described further developments of the program or planned future research on the program, which may indicate further implementation/continued use of the programs.

Thirteen of the 25 quantitative studies evaluated one or more of the four predefined elements of feasibility [30] in 6 of the 11 programs: acceptability of the burden of the program (8 studies), ease of use (8 studies), understandability of the text (4 studies), and acceptability of the program (2 studies). With the exception of one study with mixed results [57], outcomes indicated that users found the burden acceptable, the program easy to use, and the text understandable. The program was found acceptable in one study [47].

In one qualitative study, participants reported ease of use and understandability of the text as well as barriers because of confusing layout and emotive language [50]. However, the authors concluded that the program was acceptable, applicable, and understandable.

### Outcomes of the Studies on Web-Based Advance Care Planning Programs

Multimedia Appendix 3 presents an overview of the outcomes of the quantitative and qualitative studies. The 25 quantitative studies reported on evaluations of ACP as recommended by the EAPC ACP task force [1], such as the identification of goals, values, and preferences (18 studies) or documentation of preferences in a PDF output document or an AD (18 studies). Often, these aspects were part of the ACP program. In approximately half of the studies, ACP communication (13 studies), satisfaction with the program (11 studies), and ACP helpfulness (11 studies) were evaluated. Less than half of the studies evaluated ACP knowledge (11 studies) and quality of ACP/accuracy in reflecting wishes (7 studies). Few studies evaluated ACP readiness (6 studies), self-efficacy (6 studies), ACP revision over time (4 studies), (decision) concordance between the patients’ preferences and the health care professionals’ decisions (2 studies) or the health care representatives’ decisions (1 study), and health care use (1 study).

**Figure 2:**
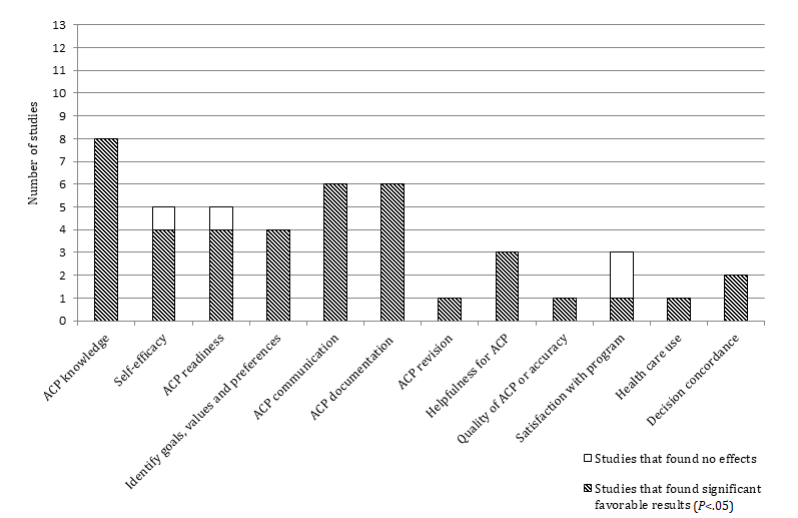
Results of quantitative studies assessing the effectiveness of Web-based advance care planning programs comparing the intervention group (Web-based advance care planning program) with baseline or control groups (N=13). ACP: advance care planning.

The research designs of 13 of the 25 quantitative studies allowed for the determination of the effectiveness of Web-based ACP programs using an RCT design and/or before and after designs. Eight studies applied an RCT design [31,39,45,49,52,54-56], and nine studies compared follow-up results of the intervention group with baseline (before and after design) [31,34,40,42,43,45,53-55] (see Multimedia Appendix 3). These studies evaluated the effectiveness of five Web-based ACP programs. Outcomes of these 13 quantitative studies indicate significantly increased ACP knowledge (8/8 studies); ACP communication (6/6 studies); ACP documentation (6/6 studies); identification of goals, values, and preferences (4/4 studies); self-efficacy (4/5 studies); and ACP readiness (4/5 studies). These outcomes are visualized in Table 3 and Figure 2 [31,34,39,40,42,43,45,49,52-56]. The remaining 12 quantitative studies were cross-sectional.

The extent to which programs were evaluated differed. For example, the programs ACP Decisions, MyICUGuide, and NVLivingWill were each evaluated considering one of the predefined outcome measures [1] in one study, whereas Making Your Wishes Known was evaluated in 12 studies in different settings considering 10 outcome measures, and PREPARE was evaluated in seven studies in different settings considering eight outcome measures.

The two qualitative studies indicated that participants were satisfied with the programs, which helped them to communicate about ACP [32,50]. In one of these qualitative studies, which evaluated the program PREPARE, participants gained more knowledge about ACP, although the section about values and beliefs was considered less relevant [50].

**Table 3:** Results of quantitative studies assessing the effectiveness of Web-based advance care planning programs comparing the intervention group (Web-based advance care planning program) with baseline and/or control groups.

Studies	Program	ACP^a^ knowledge	Self-efficacy	ACP readiness	Identification of goals, values and preferences	ACP communication	ACP documentation	ACP revision	ACP helpfulness (making decisions)	Quality of ACP or accuracy	Satisfaction	Health care use	Decision concordance
Volandes et al [31]	ACP Decisions	—^b^	—	—	—	—	**+** ^c^	—	—	—	—	↓^d^	—
Green and Levi [34]	Making Your Wishes Known	—	—	—	—	—	—	—	—	**+**	—	—	—
Markham et al [40]	Making Your Wishes Known	**+**	—	—	—	—	—	—	—	—	—	—	—
Green et al [39]	Making Your Wishes Known	**+**	—	—	**+**	**+**	—	—	**+**	—	**+**	—	—
Van Scoy et al [42]	Making Your Wishes Known	**+**	—	—	—	—	—	—	—	—	—	—	—
Levi et al [43]	Making Your Wishes Known	**+**	—	—	—	—	—	—	—	—	—	—	**+**
Green et al [45]	Making Your Wishes Known	—	NS^e^	—	—	—	—	—	—	—	—	—	**+**
Lindquist et al [49]	Plan Your Lifespan	—	—	NS	—	**+**	**+**	**+**	—	—	—	—	—
Sudore et al [53]	PREPARE For Your Care	**+**	**+**	**+**	**+**	**+**	**+**	—	—	—	—	—	—
Sudore et al [52]	PREPARE For Your Care	**+**	**+**	**+**	**+**	**+**	**+**	—	—	—	NS	—	—
Lum et al [54]	PREPARE For Your Care	**+**	**+**	**+**	—	**+**	**+**	—	—	—	—	—	—
Sudore et al [55]	PREPARE For Your Care	**+**	**+**	**+**	—	**+**	**+**	—	**+**	—	NS	—	—
Periyakoil et al [56]	The Letter Project Advance Directive	—	—	—	**+**	—	—	—	**+**	—	—	—	—
Total^f^	13	8	5	5	4	6	6	1	3	1	3	1	2

^a^ACP: advance care planning.

^b^—: Not examined by statistically comparing groups.

^c^+: significant increase.

^d^↓: significant decrease.

^e^NS: effectiveness examined, but no significant differences found.

^f^Total number of studies in which the outcome measures were examined by comparing the intervention group (Web-based advance care planning program) with baseline and/or control groups.

## Discussion

### Principal Findings

This scoping review provides an overview of Web-based, interactive, and person-centered ACP programs, including their functionalities, content, feasibility, and effectiveness.

This review identified 11—mainly developed in the United States—programs, many of which contain videos, provide tailored information, and can be used without assistance. Most of the programs contain the key elements of ACP [1], such as information about ACP, goals and preferences for future treatment and care, and included the possibility to generate a document in which patients can record their goals, values, and preferences. The extent to which programs contain functionalities such as *text-to-speech* differs. The program PREPARE, for instance, has 11 of such functionalities, whereas ACP Decisions contains five functionalities. Furthermore, the extent to which programs were evaluated differed between studies. For example, the programs ACP Decisions, MyICUGuide, and NVLivingWill were each evaluated in one study, whereas the program Making Your Wishes Known was evaluated in 12 studies in different settings.

Reportedly, programs were easy to use and not burdensome to participants. However, the feasibility of the programs was evaluated in only 13 of 27 studies for six programs, the evaluation was often limited to one or two outcome measures, and the response and completion rates were relatively low for some studies. In general, reportedly, participants were satisfied with the ACP programs. Some outcome measures, such as quality or accuracy of the program in representing wishes, health care use, its concordance with patients’ preferences, and the revision of preferences and documents over time, were less often evaluated. Overall, the studies with RCT or before and after designs comparing the intervention group with baseline or a control group showed that Web-based ACP programs are a promising approach to support patients in ACP by showing significant improvement in ACP knowledge, ACP communication, and ACP documentation.

There seems to be no link between the outcomes and the content of the programs because almost all programs address the key elements of ACP. Although many studies found results in a favorable direction, only the minority of the studies, namely, 13 of 27, use strong research designs in which groups were statistically compared.

### Comparison With Prior Research

This review focuses on providing an overview of Web-based, interactive, and person-centered ACP programs that are currently available. Although previous reviews did not have this specific focus, the reviews describe similar content and outcomes for Web-based programs related to ACP as in the this review, such as identification of preferences and treatment options, completion of ADs, the appointment of a health care representative, and they report satisfaction with the programs and increase of knowledge after using the program [14,20,21]. This review found some Web-based programs, which were also identified by the prior reviews, such as Making Your Wishes Known and PREPARE. As Butler et al [14] described, it seems that many Web-based programs in ACP, end-of-life care, and palliative care are available in the gray literature as well. For example, the interactive ACP program *My Decisions* from the United Kingdom [59] and the palliative care communication program *Tell Us* from the United States [60] are available on the internet but seemed not to be investigated in a study (when our search strategy was conducted). Therefore, the evaluation of Web-based ACP programs seems to be a challenge/opportunity for future research.

### Strengths and Limitations

This review has several strengths. We used a systematic approach, namely, the methodological framework for scoping reviews by Arksey and O’Malley [26]. The EAPC definition and recommendations for ACP allowed for a structured evaluation of the content and the effectiveness of the programs [1]. The framework of Bowen et al [30] and the CONSORT-EHEALTH checklist [29] allowed for a structured evaluation of the feasibility of the programs. The search was systematically conducted and performed with broad search terms in seven databases. Two reviewers independently screened the titles, abstracts, and full text of articles to select relevant studies.

Some limitations should also be mentioned. Importantly, it should be taken into account that the content and layout of Web-based programs are continually changing. Although the WebCite tool allowed us to archive the websites’ homepages (Multimedia Appendix 2), it is possible that (parts of the) programs have changed in the period between our review of the websites and the publication of this study. Furthermore, we only included Web-based programs which were evaluated in a study.

### Recommendations for Future Research

First, as most of the Web-based ACP programs are developed in the United States, we recommend the development of evidence-based, Web-based, interactive, and person-centered ACP programs in countries outside the United States. Ideally, to allow for proper scaling up of ACP, these programs should be tailored to local cultural and legal circumstances. To enhance the quality of Web-based ACP programs, we recommend that these Web-based ACP programs contain all key elements of ACP. Second, several important outcomes of ACP were often not reported. More clarity on which outcome measure to report, and when, would be useful. In addition, support in how to assess key outcome measures, such as concordance between preferred and received care, is needed because this important outcome measure is difficult to measure. Namely, it is not always clearly stated in medical files whether provided treatments had a curative or a palliative intent [61]. When this is not mentioned, it is difficult to determine whether treatments aligned with preferences [61]. Furthermore, it is difficult to establish a baseline measure of patients’ goals, and when goals are not documented, the concordance with these goals cannot be evaluated [62]. In addition, patients’ preferences may change during the study period, which complicates the use of this measure in practice [62]. In addition, it could be that Web-based ACP affects care in the long run, which further complicates its measurement. We recommend further research into this topic, for instance, by developing a core outcome set for ACP. Third, we recommend evaluating the feasibility of ACP programs. A clear guideline of evaluating feasibility in eHealth tools/Web-based programs is not yet available. Therefore, preferably that evaluation should be based on the framework of Bowen et al [30] and the CONSORT-EHEALTH checklist [29], which indicate important outcome measures. Fourth, we recommend the use of proper research designs, such as RCTs or before and after research designs, allowing for further determination of the effectiveness and feasibility of Web-based ACP programs. Future studies may evaluate how stakeholders other than patients perceive the role of Web-based ACP programs in the health care process, for example, general practitioners. We strongly recommend comparing the feasibility and effectiveness of the programs to ACP by health care professionals or to ACP supported by facilitators because the effectiveness of the programs in health care practice is still unknown. Finally, related to safety and technology, the safety of the generated documents by the Web-based ACP programs is still unknown, and it is also unknown whether Web-based ACP programs can be used among underserved groups who have possibly less access to these technologies, for example, patients with low eHealth or health literacy skills.

### Conclusions

This scoping review shows that Web-based, interactive, and person-centered ACP programs are mainly developed and evaluated in the United States. The Web-based programs contained the key elements of ACP, such as discussing and documenting goals and preferences for future care. In general, studies report that Web-based ACP programs tend to be feasible. Only 13 studies measured the programs’ effectiveness, and they showed significant improvement in ACP knowledge, communication, and documentation. The key outcome of ACP—concordance between preferred and received treatment and care—is yet understudied. Studies with high-quality study designs in diverse cultural contexts on feasibility and effectiveness are warranted to further establish the effectiveness of important outcomes. Furthermore, it is unknown how programs are used in practice, including attitudes of health care professionals toward Web-based ACP programs.

Overall, we conclude that Web-based, interactive, and person-centered ACP programs are promising to support patients in ACP. Web-based ACP programs may improve accessibility to ACP, allowing people to start with ACP in their own time and environment. Web-based ACP programs may, therefore, help to overcome the time and emotional barriers in the initiation of ACP and to scale up ACP.

## Appendix

Multimedia Appendix 1 Search strategy scoping review.

Multimedia Appendix 2 Links to the Web-based advance care planning programs.

Multimedia Appendix 3 Data extraction of the 27 included studies about Web-based advance care planning programs, alphabetical order of the program names and the year the article was published.

Multimedia Appendix 4 Feasibility of the Web-based advance care planning programs of the quantitative studies.

## Abbreviations

ACP: advance care planning
AD: advance directive
CONSORT-EHEALTH: Consolidated Standards of Reporting Trials of Electronic and Mobile HEalth Applications and onLine TeleHealth
EAPC: European Association for Palliative Care
eHealth: electronic health
RCT: randomized controlled trial
PREPARE: PREPARE For Your Care
