# Supplementary material for: The Feasibility and Effectiveness of Web-Based Advance Care Planning Programs: Scoping Review
Source: J Med Internet Res. 2020 Mar 17;22(3):e15578. doi: 10.2196/15578 (PMC7109619; doi:10.2196/15578)
Supplement: Multimedia Appendix 1 [file jmir_v22i3e15578_app1.docx]

Multimedia Appendix. Search strategy scoping review.

| Database | Search strategy |
| --- | --- |
| Embase.com (Embase incl. MEDLINE) | ('terminal care'/de OR 'advance care planning'/de OR 'advanced care planning'/de OR 'living will'/de OR (((terminal OR advance* OR palliative) NEAR/3 (care OR plan* OR healthcare)) OR 'end of life' OR ((advance*) NEXT/1 (directive*)) OR ((living) NEXT/1 (will*))):ab,ti) AND ('Internet'/de OR 'website'/de OR 'decision support system'/exp OR 'computer aided design'/de OR (internet OR web OR webbased OR website OR online OR ((decision) NEXT/3 (tool* OR aid* OR system* OR technique* OR program* OR instrument*)) OR ((computer) NEXT/3 (based OR aided OR design*))):ab,ti) NOT ('conference abstract')/it |
| MEDLINE Epub (Ovid) | (terminal care/ OR Advance Care Planning/ OR Living Wills/ OR (((terminal OR advance* OR palliative) ADJ3 (care OR plan* OR healthcare)) OR end of life OR ((advance*) ADJ (directive*)) OR ((living) ADJ (will*))).ab,ti.) AND (Internet/ OR Computer-Aided Design/ OR (internet OR web OR webbased OR website OR online OR ((decision) ADJ3 (tool* OR aid* OR system* OR technique* OR program* OR instrument*)) OR ((computer) ADJ3 (based OR aided OR design*))).ab,ti.) |
| PsycINFO (Ovid) | (Palliative Care/ OR Advance Directives/ OR (((terminal OR advance* OR palliative) ADJ3 (care OR plan* OR healthcare)) OR end of life OR ((advance*) ADJ (directive*)) OR ((living) ADJ (will*))).ab,ti.) AND (Internet/ OR Computer Assisted Design/ OR (internet OR web OR webbased OR website OR online OR ((decision) ADJ3 (tool* OR aid* OR system* OR technique* OR program* OR instrument*)) OR ((computer) ADJ3 (based OR aided OR design*))).ab,ti.) |
| CINAHL EBSCOhost | (MH terminal Care OR MH Advance Care Planning OR MH Living Wills OR TI (((terminal OR advance* OR palliative) N2 (care OR plan* OR healthcare)) OR end of life OR ((advance*) N1 (directive*)) OR ((living) N1 (will*))) OR AB (((terminal OR advance* OR palliative) N2 (care OR plan* OR healthcare)) OR end of life OR ((advance*) N1 (directive*)) OR ((living) N1 (will*)))) AND (MH Internet OR MH Computer Aided Design OR TI (internet OR web OR webbased OR website OR online OR ((decision) N2 (tool* OR aid* OR system* OR technique* OR program* OR instrument*)) OR ((computer) N2 (based OR aided OR design*))) OR AB (internet OR web OR webbased OR website OR online OR ((decision) N2 (tool* OR aid* OR system* OR technique* OR program* OR instrument*)) OR ((computer) N2 (based OR aided OR design*)))) |
| Cochrane Central | ((((terminal OR advance* OR palliative) NEAR/3 (care OR plan* OR healthcare)) OR 'end of life' OR ((advance*) NEXT/1 (directive*)) OR ((living) NEXT/1 (will*))):ab,ti) AND ((internet OR web OR webbased OR website OR online OR ((decision) NEXT/3 (tool* OR aid* OR system* OR technique* OR program* OR instrument*)) OR ((computer) NEXT/3 (based OR aided OR design*))):ab,ti) |
| Web of Science | TS=(((((terminal OR advance* OR palliative) NEAR/2 (care OR plan* OR healthcare)) OR "end of life" OR ((advance*) NEAR/1 (directive*)) OR ((living) NEAR/1 (will*)))) AND ((internet OR web OR webbased OR website OR online OR ((decision) NEAR/2 (tool* OR aid* OR system* OR technique* OR program* OR instrument*)) OR ((computer) NEAR/2 (based OR aided OR design*)))) ) AND DT=(article) |
| Google Scholar | "terminal\|advance\|palliative care\|plan\|healthcare"\|"end of life"\|"advance directive"\|"living will" internet\|web\|webbased\|website\|online\|"decision tool\|aid\|system\|technique\|program\|instrument"\|"computer based\|aided\|design" |
