# Supplementary material for: The Feasibility and Effectiveness of Web-Based Advance Care Planning Programs: Scoping Review
Source: J Med Internet Res. 2020 Mar 17;22(3):e15578. doi: 10.2196/15578 (PMC7109619; doi:10.2196/15578)
Supplement: Multimedia Appendix 2 [file jmir_v22i3e15578_app2.docx]

Multimedia Appendix. Links to the Web-based advance care planning programs.

| ACP Decisions | http://www.ACPdecisions.org/patients/ Archived by WebCite® at http://www.webcitation.org/6zyQpKfhS accessed: 2018-06-06 |
| --- | --- |
| Death over Dinner | http://deathoverdinner.org/  Archived by WebCite® at http://www.webcitation.org/6zyRKQdnH accessed: 2018-06-06 |
| Five Wishes | https://www.agingwithdignity.org/five-wishes/about-five-wishes  Archived by WebCite® at http://www.webcitation.org/6zySif2B3 accessed: 2018-06-06 |
| Making Your Wishes Known | https://www.makingyourwishesknown.com/ Archived by WebCite® at http://www.webcitation.org/6zySwaoAU accessed: 2018-06-06 |
| MyDirectives | https://www.mydirectives.com/  Archived by WebCite® at http://www.webcitation.org/6zySoHDtB accessed: 2018-06-06 |
| MyICUGuide | https://www.myicuguide.ca/  Archived by WebCite® at http://www.webcitation.org/6zyT9hNs6 accessed: 2018-06-06 |
| NVLivingWill | http://www.nvlivingwill.com/  Archived by WebCite® at http://www.webcitation.org/6zyTAhDmC accessed: 2018-06-06 |
| Plan your Life Span | http://www.planyourlifespan.org/ Archived by WebCite® at http://www.webcitation.org/6zyTBiZZ4 accessed: 2018-06-06 |
| PREPARE For Your Care | https://prepareforyourcare.org/  Archived by WebCite® at http://www.webcitation.org/6zyQuuaRg accessed: 2018-06-06 |
| The Letter project Advance Directive | https://med.stanford.edu/letter/advancedirective.html Archived by WebCite® at http://www.webcitation.org/6zyTCzvLn accessed: 2018-06-06 |
| Think Ahead | www.thinkahead.ie Archived by WebCite® at http://www.webcitation.org/6zyTRe3ds accessed: 2018-06-06 |
