# Supplementary material for: The Feasibility and Effectiveness of Web-Based Advance Care Planning Programs: Scoping Review
Source: J Med Internet Res. 2020 Mar 17;22(3):e15578. doi: 10.2196/15578 (PMC7109619; doi:10.2196/15578)
Supplement: Multimedia Appendix 3 [file jmir_v22i3e15578_app3.docx]

Multimedia Appendix. Data extraction of the 27 included studies about Web-based advance care planning programs, alphabetical order of the program names and the year the article was published.

| Program, first author, year (country) | Participants + setting | Study design, intervention and measures | Results of the studies (feasibility [29, 30] and effectiveness [1]) |
| --- | --- | --- | --- |
| - ACP Decisions  - Volandes, 2016 (USA) [31] | - Participants: hospitalized patients with late-stage disease (N = 3119) - Setting: one hospital, one hospice and among 30 primary care physicians | - Controlled before-after study design  - In intervention region: 1-4-h training of physicians in ‘ACP Decisions’ - In control region: no training of physicians | ACP documentation: Patients in intervention region: increase from 3% (11/346 participants) before the intervention to 40% (1,107/2,773 participants) after the intervention (*P* < .001).  Primary care providers (of patients > 75 years) in the intervention region: 37% (1,437/3,888) compared to the control region 26% (10,760/42,099) (*P* < .001).  Healthcare use: Intervention region: discharge from hospital to hospice increased from 6% to 14% (*P* < .001). Average insurance costs per patient in last month of life were $3,458 lower in the intervention region than in the control region. |
| - Death over Dinner  - Lambert South, 2017 (USA) [32] | - Participants: adults (N = 240) - Setting: home of participant or research assistant, or restaurant | - Qualitative research design - 52 Dinner conversations (similar to focus groups) | ACP communication:  Three major themes emerged: desire for a good death, tactics for coping, and topics that elicit fear or uncertainty.  Satisfaction:  Participants were satisfied; based on their positive feedback, the authors concluded the program is a promising tool for families to engage in end-of-life conversations. |
| - Five Wishes  - Chovan, 2007 (USA) [33] | - Participants: residents of nursing homes (N = 29) - Setting: two nursing homes | - Prospective cohort study design - ≥5 supported sessions per participant to complete program  - Follow-up review: questions (Werth & Blevins, 2002) | Identification of goals, values, preferences / ACP documentation: Participants (N = 15) completed the program, and indicated preferences in a generated AD. Seven participants completed parts and seven participants only showed interest in the introductory phases. |
| - Making Your Wishes Known  - Green, 2009 (USA) [34] | - Participants: study 1: adults (N = 50), study 2: patients with cancer (N = 34)  - Setting: study 1: internal medicine outpatient practice, study 2: clinics at a medical center | - Cross-sectional study design (however, after post-editing program some outcomes were examined again) - Two pilot tests examined participants’ use of the ACP program | Burden: Study 1: participants spent on average 106 min completing the program, and indicated this was not burdensome.  Ease of use: Preliminary testing showed participants found the program easy to use *(No numbers described in article)*.  ACP knowledge / ACP helpfulness: Study 1: participants were satisfied with how the program improved their knowledge and helped them to make decisions: 4.2 (1 = very dissatisfied, 5 = very satisfied).  Identification of goals, values and preferences: All participants of both studies indicated their preferences in the program in a generated AD.  Quality of ACP / Accuracy reflection wishes:  Study 2: the program was accurate in representing wishes: increased from 5.5 to 6.5 after post-editing the program, *P* < .001 (1 = not at all accurate, 7 = very accurate).  Satisfaction:  Both studies: participants were satisfied with the program: 8.5 (1 = not at all satisfied, 10 = extremely satisfied). |
| - Making Your Wishes Known  - Hossler, 2011 (USA) [35] | - Participants: patients with ALS (N = 17) - Setting: medical center | - Before-after study design (however, extracted outcome measures were only assessed after) - Participants’ use of the ACP program was examined - Surveys: Satisfaction with ACP; decisional conflict (O’Connor, 1995); Satisfaction with decision (Holmes-Rovner et al., 1996); Time and Effort | Burden: Participants (N = 6) reported no burden for completion. Participants judged the amount of information appropriate: 6.8 (1 = too little, 5 = about right, 10 = too much).  ACP knowledge: The program helped participants to increase knowledge about ACP: 4.3 (1 = low, 5 = high).  Identification of goals, values and preferences: The program helped participants to put wishes into words: 4.2, helped to clarify wishes regarding healthcare: 4.3, and helped to clarify values: 4.1 (1 = low, 5 = high).  ACP communication: The program prepared participants to discuss wishes with family: 4.2 and doctors: 4.1 (1 = low, 5 = high).  ACP documentation: All participants completed the program and indicated their preferences in a generated AD.  ACP helpfulness: The program helped to make end-of-life care decisions: 4.1, choosing a spokesperson: 3.9, and helped to feel control about future health: 4.3 (1 = low, 5 = high).  Quality of ACP / Accuracy reflection wishes: The program was accurate in reflecting wishes: 8.6 (1 = not at all accurate, 10 = extremely accurate).  Satisfaction: Participants reported a high level of satisfaction: 8.5 (1 = not at all satisfied, 10 = extremely satisfied), and judged the program positively: 4.2 (1 = very dissatisfied, 5 = very satisfied). |
| - Making Your Wishes Known  - Levi, 2011 (USA) [36] | - Participants: patients who previously created an AD using program (N = 19), physicians: no prior knowledge of the patients (N = 14)  - Setting: medical center | - Vignette design: pilot study on responses to vignettes in program - Physicians reviewed ADs, made treatment decisions for end-of-life scenarios  - Patients indicated how well physicians translated wishes into clinical decisions | Decision concordance: patient-physician / Accuracy reflection wishes: Level of concordance was 84%. Patients’ rating of how well physicians translated their AD into medical decisions was 8.4 (1 = extremely poor, 10 = extremely well). Physicians’ overall rating of their confidence at accurately translating patients’ wishes into clinical decisions was 7.8 (1 = not at all confident, 10 = extremely confident). |
| - Making Your Wishes Known  - Schubart, 2012 (USA) [37] | - Participants: adults of 30 years or older (N = 24)  - Setting: recruitment in senior center | - Prospective cohort study design - Participants completed program twice, 4-6 weeks apart - Responses on questions in program were examined | Identification of goals, values, preferences / ACP documentation: All participants completed the program and indicated preferences in a generated AD. The General Wishes and Quality of Life statements remained consistent over time, Specific Wishes did not remain consistent.  ACP communication: Participants (N = 16) shared their AD with others. |
| - Making Your Wishes Known  - Markham, 2015 (USA) [40] | - Participants: African American adults (N = 18) - Setting: 5 predominantly African American churches | - Before-after study design (pilot study) - Participants’ use of the ACP program was examined - Surveys: ACP Knowledge (Green & Levi, 2011); Satisfaction with decision (Holmes-Rovner et al., 1996), Satisfaction with ACP, Accuracy of advance directive, Time and Effort - Follow up phone interviews after a few weeks | Burden: Participants spent on average 60-90 min on the program, reporting low burden: 1.3 (1 = no burden, 4 = extreme burden).  ACP knowledge:  Increased from 45% to 61%, *P* < .001.  Identification of goals, values, preferences / ACP helpfulness: Participants reported that the program helped them clarify their healthcare wishes and feel greater control (88%).  ACP communication: The program prepared participants to discuss their wishes with physicians: 94%, and family members: 100%. Follow-up phone interviews: >80% shared their AD with relatives.  ACP documentation:  All participants completed the program indicating their preferences in a generated AD.  ACP revision: None of the participants reported having changed their spokesperson, one reported changing wishes.  Quality of ACP / Accuracy reflection wishes**:**   The program accurately reflected wishes: 6.4 (1 = not at all accurate, 7 = extremely accurate).  Satisfaction:  Participants were satisfied with the ACP process: 9.4 (1 = not at all satisfied, 10 = extremely satisfied) and with decisions made: 10.0 (6 = very satisfied, 30 = not satisfied). |
| - Making Your Wishes Known  - Green, 2015 (USA) [39] | - Participants: patients with advanced cancer (N = 200)   - Setting: tertiary care academic medical center | - Randomized controlled trial design - Intervention group: ACP program - Control group: educational materials, online state-approved AD - Participants’ use of the ACP program was examined - Surveys: ACP knowledge (internally validated); Satisfaction ACP process (developed for study) | Burden: The intervention group spent more time on the program, on average 70 min (range 15-120 min) compared to 26 min in the control group (10-65 min), *P* < .001. There were no reported adverse effects.  ACP knowledge: The intervention group increased with 13%, the control group increased with 4%, *P* < .01.  Identification of goals, values, preferences / ACP communication / ACP helpfulness / Satisfaction: Participants were satisfied with the ACP process in both groups (overall score: quality of information, helpfulness in values clarification, helpfulness in decision making, and helpfulness in communicating wishes). Scores were higher in the intervention group: 51.2, than the control group: 48.4, *P* < .01 (12 = low, 60 = high).  ACP documentation: Participants (N = 198) completed the program, indicating their preferences in a generated AD. |
| - Making Your Wishes Known  - Schubart, 2015 (USA) [41] | - Participants: patients with advanced cancer undergone initial curative intent surgery (N = 48), non-curative intent surgery (N = 31)  - Setting: academic medical center | - Retrospective cohort study design - Review of data from a RCT examining the use of the program  - Review of medical records | Identification of goals, values, preferences: Patients (N = 79) had indicated their preferences in a generated AD, which were analyzed in this study. Patients who underwent curative intent surgery indicated in their treatment wishes that they did not prefer more aggressive end-of-life treatments compared to patients whose treatment was non-curative intent. |
| - Making Your Wishes Known  - Van Scoy, 2016 (USA) [42] | - Participants: patients with heart failure (N = 24), patients with COPD (N = 25) - Setting: 2 academic university medical intensive care units | - Before-after study design - Participants’ use of the ACP program was examined -Surveys: ACP Knowledge (face valid); Satisfaction with Decision aid (designed to evaluate program, face-valid, Green et al., 2014; Markham et al., 2014); Accuracy (designed to evaluate program, Markham et al., 2014); Decisional satisfaction (Holmes-Rovner, 1996); Decisional conflict (O’Connor, 1995) | ACP knowledge:  ACP knowledge scores increased with 18%, *P* < .001.  Identification of goals, values, preferences / ACP documentation: Participants (N = 49) completed the program indicating their wishes in a generated AD. 90% reported the program was helpful in values clarification.  ACP communication: 92% reported the program helped communicating wishes.  ACP revision: 31% felt the need to edit the initial AD.  ACP helpfulness: 92% reported the program was helpful in decision-making.  Quality of ACP / Accuracy reflection wishes: > 90% reported the AD was highly accurate.  Satisfaction: > 90% reported being satisfied across all domains, and on overall satisfaction with decisions: 26.1 (6 = low, 30 = high). |
| - Making Your Wishes Known  - Schubart, 2017 (USA) [44] | - Participants: adults (N = 33) - Setting: Medical Center | - Prospective cohort study design -Responses on questions in program were examined - Participants completed program at 3 visits, 2 weeks apart | Identification of goals, values, preferences / ACP documentation: All participants completed the program 3 times and indicated their wishes in a generated AD. The program was reliable in representing General Wishes for treatment (94% selected identical response each visit) and moderately for Specific Wishes for Treatment (Cronbach α > .90). |
| - Making Your Wishes Known  - Levi, 2017 (USA) [43] | - Participants: patients with ALS (N = 44), clinicians who treated the patients (N = 15) - Setting: interdisciplinary ALS clinic | - Before-after study design - Participants’ use of the ACP program was examined - Before and 3 months after intervention, clinicians reviewed clinical vignettes and made treatment decisions. Patients indicated agreement with these decisions - Surveys: ACP Knowledge pre- and post-intervention (Green et al., 2015); Quality of Life ALS-Specific (Simmons et al., 2006); Satisfaction with ACP; Decisional Conflict (O’Connor, 1995); Satisfaction with Decision (Holmes-Rovner, 1996); Time and Effort | Burden: Participants spent on average 77 min on the program, none reported burden for completion.  ACP knowledge:  Scores increased from 48% to 66% correct responses, *P* = .001.  Identification of goals, values, preferences / ACP communication / ACP documentation / Satisfaction: All but one patient (N = 43) completed the program and indicated wishes in a generated AD. Patients reported a high level of overall satisfaction with the program: 52.7 (12 = low, 60 = high) including satisfaction in clarifying values, preparing to discuss wishes and choosing a spokesperson.  ACP helpfulness: Patients reported a high level of satisfaction with decisions: 26.4 (6 = low, 30 = high) and low level of decisional conflict: 28.8 (20 = low, 80 = high).  Quality of ACP / Accuracy reflection wishes: Patients reported the generated AD accurately reflected their wishes: 9.4 (1 = low, 10 = high).  Decision concordance: Concordance between patients’ preferences and the clinicians decisions significantly increased from 52% to 92%, *P* < .001. Confidence of clinicians that decisions accurately represented patients’ wishes increased from 3.3 to 6.5, *P* = .001 (1 = low, 10 = high). |
| - Making Your Wishes Known,  -MyDirectives,  - PREPARE For Your Care  - Holland, 2017 (USA) [38] | - Participants: community dwelling adults with multiple chronic health conditions (N = 40)  - Setting: primary care clinic | - Prospective comparative study design (pilot study) - Nurse-supported scripted discussions - Groups: participants completed 1 of 4 programs (MYWK, PREPARE, MyDirectives, and educational ACP booklet) - ACP engagement survey (pre-and post-intervention) | ACP knowledge: 5-point scale scores for MYWK: increased from 3.9 to 4.4, PREPARE: increased from 3.5 to 4.3, MyDirectives increased from 3.2 to 4.2, AHCP booklet: increased from 3.4 to 4.8.  Self-efficacy: 5-point scale scores for MYWK: increased from 4.1 to 4.6, PREPARE: increased from 4.1 to 4.5, MyDirectives: increased from 3.3 to 4.5, AHCP booklet: increased from 3.9 to 4.7.  ACP readiness: 5-point scale scores for MYWK: increased from 3.9 to 4.8, PREPARE: increased from 4.1 to 4.6, MyDirectives: increased from 3.4 to 4.6, AHCP booklet: increased from 3.9 to 4.9.  Identification of goals, values, preferences:  The programs helped to clarify values: 5-point scale scores for MYWK: = 4.6, PREPARE: 4.6, MyDirectives: 4.1, AHCP booklet: 4.3 and wishes: MYWK: 4.6, PREPARE: 4.4, MyDirectives: 4.2, AHCP Booklet: 4.5.  ACP communication: The programs helped to put wishes into words: 5-point scale scores for MYWK: 4.0, PREPARE: 4.4, MyDirectives: 4.3, AHCP booklet: 4.5, helped to prepare for discussions with doctors: MYWK: 4.1, PREPARE: 3.9, MyDirectives: 4.1, AHCP booklet: 4.3 and family: MYWK: 4.4, PREPARE: 4.3, MyDirectives: 4.4, AHCP booklet: 4.5.  ACP documentation:  Participants (N = 34 of 40) completed or updated an AD; all participants (N = 40) identified a healthcare representative.  ACP helpfulness: The programs helped to make important end-of-life healthcare decisions: 5-point scale scores for MYWK: 4.1, PREPARE: 4.6, MyDirectives: 4.6, AHCP booklet: 4.5.  Quality of ACP / Accuracy reflection wishes: 10-point scale scores for MYWK: 9.3, PREPARE: 8.6, MyDirectives: 8.7, AHCP booklet: 8.7) (1 = not at all accurate, 10 = extremely accurate).  Satisfaction: Participants were overall satisfied with the program on aspects described above: 5-point scale scores for MYWK: 4.3, PREPARE: 4.4, MyDirectives: 4.3, AHCP booklet: 4.4 and with the program: 10-point scale scores for MYWK: 8.2, PREPARE: 8.5, MyDirectives: 9.0, AHCP booklet: 8.3 (1 = not at all satisfied, 10 = extremely satisfied). |
| - Making Your Wishes Known  - Green, 2018 (USA) [45] | - Participants: dyads of patients with advanced illness and family members (healthcare representative) (N = 285)  - Setting: two tertiary care medical  centers | - Randomized controlled trial (2x2)  - Intervention group: ACP program  - Control group:  Basic ACP: online state-approved AD and brochure  - Programs were completed alone or with the family member  - Surveys: pre–post self-efficacy in family members (Nolan et al, 2009); concordance between patients and family members using clinical vignettes | Self-efficacy:  Family members’ average scores increased for MYWK from 90.2 to 92.1 for the intervention group (*P* = .13), and from 90.1 to 93.3 in the control group (*P* = .004) (0 = low, 100 = high). Average scores increased from 90,2 to 92.6, (*P* = .03) for patients who completed the program alone and from 90.1 to 92.8 (*P* = .03) for patients who completed the program together with their family member.  Decision concordance: patient / healthcare representative The adjusted concordance score was significantly higher in MYWK (85%) than in basic ACP (79.7%), *P* = .03, with no between-group difference. There were no significant differences between patients who completed the program alone and patients who completed the program with their family member. |
| - MyDirectives  - Fine, 2016 (USA) [46] | - Sample (N = 900) of program users  - Setting: unspecified | - Descriptive research study design - Responses on questions in program were examined | Identification of goals, values, preferences: Users (N = 900) indicated preferences in the program, which generated an AD.  ACP revision:  12% changed their AD at least once (range 1 to 4). 75% of changes were made more than 1 day after AD creation, with 30% of changes made from 4 months after AD creation. |
| - MyICUGuide  - Van Scoy, 2017 (USA) [47] | - Participants: family members of ICU-patients with critical illness (N = 27) - Setting: Intensive Care Unit room | - Cross-sectional study design - Assessment questionnaire (informed by framework for evaluation of sensibility (Feinstein, 1987)) measuring clarity of language, length, ease of use and understanding, helpfulness; burden of study involvement - Open-ended interview questions | Burden: Participants had an average score of 1.67 (1 = not burdensome at all, 10 = extremely burdensome).  Ease of use: 93% had a rating of 4 or 5 (1 = very difficult, 5 = very easy).  Understandability of the text: 100% rated usefulness as 4 or 5 (1 = very unclear, 5 = very clear). 55% suggested revisions (eliminating areas of redundancy, word changes, etc.).  Acceptability: 96% had a rating of 4 or 5 (1 = definitely would not recommend, 5 = definitely would recommend).  ACP helpfulness: 93% of family members rated the program helpful for thinking about treatments for serious illness.  Satisfaction:  100% rated overall experience and readability positively with 4 or 5 (5-point scale). In the interview questions family members noted that the program provided engaging and empowering information for decision making. |
| - NVLivingWill  - Klugman, 2013 (USA) [48] | - Participants: unspecified program users (N = 90)  - Setting: unspecified | - Cross-sectional study design - Responses on questions in program were examined - Evaluation survey | Ease of use: Ease of use was the most common reason to choose for the program (65%).  Identification of goals, values, preferences / ACP documentation: Program users (N = 90) completed the program indicating their wishes in a generated AD. |
| - Plan your Life Span  - Lindquist, 2017 (USA) [49] | - Participants: adults older than 65 years (N = 385) - Setting: recruited by community-based patient/ stakeholder partners, community centers, waiting rooms, senior centers | - Randomized controlled trial - Intervention group: ACP program - Control group: control website - Survey: planning behavior and communication of plans, perception of importance of planning - 1 and 3 month phone follow up | ACP readiness: Perception of importance of planning had an overall non-significant increase in the intervention group at both follow-up time points compared to the control group: 0.60, *P* = .08 and 0.56, *P* = .11 (1 = not at all important, 5 = completely important).  ACP communication / ACP documentation / ACP revision: Planning behavior (making a plan) and ACP communication scores increased 0.22 points more in the intervention group compared to the control group between baseline and after 1 month (1 = strongly disagree, 5 = strongly agree). Planning behavior and ACP communication at one month increased more in the intervention group than in the control group: 1.25 points more, *P* = .005 (1 = strongly disagree, 5 = strongly agree). Results were similar after 3 months (*P* = .04). |
| - PREPARE For Your Care  - Sudore, 2014 (USA) [53] | - Participants: adults: ethnically diverse and age > 60 (N = 43) - Setting: three low-income senior centers | - Before-after study design - Participants’ use of the ACP program was examined - Surveys (after 1 week): ACP Engagement (Sudore et al., 2013); ACP behavior change (Fried et al., 2012); Ease of understanding and Usefulness in ACP (Sudore et al., 2007); Satisfaction, Ease of use | Ease of use: The program was rated as easy to use: 9 (10-point scale).  Understandability of the text: The program was rated as easy to understand: 4.2 (5-point scale).  ACP knowledge: Scores increased from 3.7 to 4.3, *P* < .001 (5-point scale).  Self-efficacy:  Scores increased from 3.7 to 4.2, *P* < .001 (5-point scale).  ACP readiness: Scores increased from 2.8 to 3.4, *P* < .001 (5-point scale).  Identification of goals, values, preferences / ACP communication / ACP documentation: Action measures (choose decision-maker and flexibility, decide what is important in life and for medical care, talk with doctors and ask doctors questions, sign forms about preferences and a healthcare representative): no significant change before and one week after intervention: 7.7 at baseline, to 7.7 after one week, *P* = .56. Precontemplation decreased for most actions: 61% to 35%, *P* < .003, average decrease: 21% (range 16%-33%).  ACP helpfulness: The program was rated as useful: 4.2 (5-point scale).  Satisfaction: Participants were satisfied with the program: 9 (10-point scale). |
| - PREPARE For Your Care  - Ouchi, 2017 (USA) [51] | - Participants, 65 years or older (N = 24) - Setting: recruited from cohort survey about Geriatric emergency department care | - Cross-sectional study design - Participants’ use of the ACP program was examined - Surveys: ease of use | Ease of use:  Participants rated the program easy to use for themselves: 8.4, and others: 7.3 (1 = very hard, 10 = very easy).  Identification of goals, values, preferences:  71% completed one or more of 5 modules (choosing a healthcare representative, deciding what matters most, deciding leeway for healthcare representatives, communicate wishes, ask doctors questions). |
| - PREPARE For Your Care  - Sudore, 2017 (USA) [52] | - Participants: adults of 65 years or older (N = 414) - Setting: emergency department | - Randomized controlled trial design - Intervention group: ACP program + easy-to-read advance directive - Control group: easy-to-read advance directive - Surveys: ACP engagement at 1 week, 3 months and 6 months (Sudore et al., 2013); Satisfaction, Ease of Use | Ease of use: No significant differences between intervention group and control group, scores were high in both groups: 9.0 vs 8.7, *P* = .31 (10-point scale).  ACP knowledge / self-efficacy / ACP readiness / ACP communication:  ACP engagement increased at each follow-up, *P* < .001, including higher process (e.g. knowledge, self-efficacy, readiness) and action (e.g. ACP communication) scores.  Identification of goals, values and preferences / ACP documentation: Participants (N = 384) completed the program, indicating their preferences in a generated AD. ACP documentation was higher in the intervention group than in the control group after 6 months: 35% vs 25%, *P* = .04.  Satisfaction: No differences between intervention and control group, including comfort reviewing the program: 4.5 vs 4.4, *P* = .57, and helpfulness: 4.4 vs 4.3, *P* = .19 (5-point scale). |
| - PREPARE For Your Care  - Cresswell, 2018 (USA) [50] | - Participants: patients with cancer (N = 22) - Setting: ambulatory cancer center | - Qualitative research design - Participants’ use of the ACP program was examined - Cognitive interviews followed by a brief semi-structured interview | Burden: Some participants had difficulty with some text about end of life in the program since this was emotional for them.  Ease of use: Participants appreciated the ease of navigating through the program, sometimes they thought the layout was confusing.  Understandability of the text: Participants found the language in the program understandable, clear and appropriate.  ACP knowledge: Participants engaged in a reflective process while reviewing the program and simultaneously increased ACP knowledge.  Identification of goals, values, preferences: Responses to the section of clarifying values and beliefs about what matters most in life in the program were mixed, some participants thought some content was less relevant.  ACP communication: The program stimulated thinking about naming a decision-maker and the importance of talking about future care.  Satisfaction: Participants agreed PREPARE was acceptable and applicable for cancer patients. |
| - PREPARE For Your Care  - Lum, 2018 (USA) [54] | - Participants: patients of 60 years or older with two comorbidities (N = 414) - Setting: primary care | - Randomized controlled trial design - Intervention group: ACP program + easy-to-read advance directive - Control group: easy-to-read advance directive - Surveys: ACP engagement at baseline, 1 week, 3 months and 6 months (Sudore et al., 2013); | ACP knowledge:  Scores of the intervention group increased more (16%) than the control group (9%), *P* < .001.  Self-efficacy: Scores of the intervention group increased more (11%) than the control group (2.5%), *P* < .001.  ACP readiness: Scores of the intervention group increased more (12%) than the control group (8.6%), *P* = .005.  ACP communication: Both groups significantly increased in engagement in discussions measures with greater increases for the intervention group (99.5%) than for the control group (93.3%), *P* < .001.  ACP documentation: Both groups significantly increased in documentation with greater increases for the intervention group (99.5%) than for the control group (90.4), *P* < .001. |
| - PREPARE For Your Care  - Sudore, 2018 (USA) [55] | - Participants: English or Spanish speaking patients of 55 years or older with 2 or more chronic or serious illnesses (N = 986) - Setting: 4 primary care clinics | - Randomized controlled trial design  - Intervention group: ACP program + easy-to-read advance directive - Control group: easy-to-read advance directive - Surveys: Ease of use, Satisfaction, Helpfulness (Sudore, 2016); new ACP documentation medical record 15 months after enrollment; ACP Engagement Survey at baseline, 1 week, 3 months, 6 months, 12 months (Sudore, 2013; Sudore, 2017) | Ease of use: Ease of use was high in the intervention group (8.1) and the control group (8.0), *P* = .46 (1 = very hard, 10 = very easy).  ACP knowledge / self-efficacy / ACP readiness: More participants in the intervention group improved behavior change scores (469 of 481; 98%) than the control group (441 of 505; 87%), *P* < .001. More participants in the intervention improved action scores (456 of 481; 95%) than the control group (396 of 505; 78%), *P* < .001.  ACP communication: More participants in the intervention group improved behavior change scores (464 of 481; 97%) than the control group (430 of 505; 85%), *P* < .001, More participants in the intervention improved action scores (451 of 481; 94%) than the control group (397 of 505; 79%), *P* < .001.  ACP documentation: Documentation at 15 months was higher  in the intervention group (207/481 participants; 43%) than the control group (167/505; 32%), *P* < .001). More participants in the intervention group improved behavior change scores (472/481; 98%) than the control group (419 of 505; 83%), *P* < .001. More participants in the intervention improved action scores (422/481; 88%) than the control group (237/505; 47%), *P* < .001.  ACP helpfulness: Participants in the intervention group perceived the program more helpful (4.4) than the control group (4.2), *P* < .001 (1= not at all, 5 = extremely).  Satisfaction: Comfort viewing was high in the intervention group (4.1) and the control group (4.0), *P* = .37. Both intervention (4.2) and control group (4.1) would recommend the program, *P* = .36 (1= not at all, 5 = extremely). |
| - The Letter project advance directive  - Periyakoil. 2017 (USA) [56] | - Participants: adults of 40 years or older (N = 400) - Setting: project link was posted on Letter Project portal and disseminated on social media; electronic list serves | - Randomized controlled trial design - Intervention group: ACP program - Control group: traditional advance directive  - Survey: survey acceptability and usefulness in capturing values and preferences was formulated after discussions with multiethnic older adults and clinicians | Burden: The intervention group was more comfortable using the program than the control group: 89% vs 73%, *P* < .001.  Understandability of the text: The intervention group found the program easier to read and understand: 1.5, than the control group: 2.5, *P* < .001 (1 = strongly agree, 5 = strongly disagree).  Identification of goals, values, preferences / ACP documentation: All participants completed the program, indicating preferences in a generated AD. The program better helped to reflect what matters most, in the intervention group: 1.6, than the control group: 2.2, *P* < .001, and better stimulated thinking about end of life care and treatment in the intervention group: 1.6, than the control group: 2.2, *P* < .001 (1 = strongly agree, 5 = strongly disagree).  ACP helpfulness: The program better helped describe how participants make medical decisions in their family in the intervention group: 1.7, than in the control group: 2.2, *P* < .001. The program could help their doctors better understand end-of-life preferences in the intervention group: 1.6, than in the control group: 2.0, and for their families: 1.6 in the intervention group: 2.0, than the control group, *P* < .001. |
| - Think Ahead  - O'Shea, 2014 (Ireland) [57] | - Participants: clinically stable patients, between 40-70 years old (N = 100) - Setting: primary care setting | - Cross-sectional study design - Participants’ use of the ACP program was examined - Telephone surveys after 1 and after 3 weeks | Burden: 74% reported completing the program did not cause upset.  Understandability of the text: 37% indicated some difficulty in completion due to difficulties in understandability of the text.  Ease of use:  63% indicated ‘no difficulty’ in the completion.  Acceptability: 68% indicated they felt the program would be of general interest, 87% indicated it should be more widely available.  Identification of goals, values and preferences / ACP documentation: Participants indicated their preferences completing the whole document (29 of 92 participants), or parts (41%).  ACP communication:  83% was encouraged to discuss preferences with relatives. |
