# Supplementary material for: The Feasibility and Effectiveness of Web-Based Advance Care Planning Programs: Scoping Review
Source: J Med Internet Res. 2020 Mar 17;22(3):e15578. doi: 10.2196/15578 (PMC7109619; doi:10.2196/15578)
Supplement: Multimedia Appendix 4 [file jmir_v22i3e15578_app4.docx]

Multimedia Appendix. Feasibility of the Web-based advance care planning programs of the quantitative studies, based on Bowen et al. [30] and the CONSORT-EHEALTH checklist [29].

| First author, year | Program | Participation rate % of contacted participants | Completion rate: % of those who provided consent completing entire program | Burden acceptable | Ease of use | Understandability of the text in the program | Acceptability | Log data of users described | User feedback obtained | Further developments / research of program described |
| --- | --- | --- | --- | --- | --- | --- | --- | --- | --- | --- |
| Volandes, 2016 | ACP Decisions |  | 37% (1,437/  3,888) - 40% (1,107/  2,773)^a^ |  |  |  |  | no | no | yes |
| Chovan, 2007 | Five Wishes | 73% (29/40) | 52% (15/29) |  |  |  |  | no | yes | no |
| Green, 2009 | Making Your Wishes Known | 100% (34/34 + 50/50) | 100% (34/34 + 50/50) | **+** | **+** |  |  | no | no | yes |
| Hossler, 2011 | Making Your Wishes Known |  | 100% (17/17) | **+** |  |  |  | no | no | yes |
| Levi, 2011 | Making Your Wishes Known | 77% (20/26) | 95% (19/20) |  |  |  |  | no | yes | yes |
| Schubart, 2012 | Making Your Wishes Known | 43% (29/67) | 83% (24/29) |  |  |  |  | no | no | yes |
| Markham, 2015 | Making Your Wishes Known | 44% (18/41) | 100% (18/18) | **+** |  |  |  | no | yes | no |
| Green, 2015 | Making Your Wishes Known | 14%  (200/ 1447) | 99%  (198/200) | **+** |  |  |  | no | no | no |
| Schubart, 2015 | Making Your Wishes Known |  |  |  |  |  |  | no | no | yes |
| Van Scoy, 2016 | Making Your Wishes Known |  | 100% (49/49) |  |  |  |  | no | no | yes |
| Schubart, 2017 | Making Your Wishes Known | 57% (36/63) | 92% (33/36) |  |  |  |  | no | no | no |
| Levi, 2017 | Making Your Wishes Known | 39%  (50/ 130) | 86% (43/50)% | **+** |  |  |  | no | no | no |
| Holland, 2017 | MYWK, MyDirectives, PREPARE For Your Care | 61% (40/66) | 95% (38/40) |  |  |  |  | no | no | no |
| Green, 2018 | Making Your Wishes Known | 19%  (285/ 1536) | 94% (267/285) |  |  |  |  | no | no | yes |
| Fine, 2016 | MyDirectives |  |  |  |  |  |  | no | no | yes |
| Van Scoy, 2017 | MyICUGuide | 58% (36/62) | 72% (26/36) | **+** | **+** | **+** | **+** | yes | yes | yes |
| Klugman, 2013 | NVLivingWill, |  |  |  | **+** |  |  | no | yes | no |
| Lindquist, 2017 | Plan your Life Span | 82%  (385/470) | 99,7% (384/385) |  |  |  |  | no | no | no |
| Sudore, 2014 | PREPARE For Your Care |  | 100% (43/43) |  | **+** | **+** |  | no | yes | yes |
| Ouchi, 2017 | PREPARE For Your Care | 39% (24/61) | 71% (17/24)^a^ |  | **+** |  |  | no | no | no |
| Sudore, 2017 | PREPARE For Your Care | 44%  (414/938) | 99,8% (413/414) |  | **NS** |  |  | no | no | no |
| Lum, 2018 | PREPARE For Your Care | 44%  (414/938) | 99,8% (413/414) |  |  |  |  | no | no | no |
| Sudore, 2018 | PREPARE For Your Care | 55%  (986/1797) | 100% (986/986) |  | **NS** |  |  | no | no | yes |
| Periyakoil, 2017 | The Letter Project Advance Directive | 52%  (400/770) | 100% (400/400) | **+*** |  | **+*** |  | no | no | no |
| O'Shea, 2014 | Think Ahead | 92%  (92/100) | 31% (29/92) | **+/-** | **+** | **+/-** | **+/-** | no | yes | no |
| Total number of studies | 25 |  |  | 8 | 8 | 4 | 2 | 1 | 7 | 12 |
| Legend:  ^a^ = completed at least 1 module  + = high score  - = low score  +/- = mixed results  +* = significant increase  NS = significance examined, but no significant differences found  Empty cell = not examined  yes = Addressed in program, no = Not addressed in program | | | | | | | | | | |
